# An outbreak of intestinal schistosomiasis, alongside increasing urogenital schistosomiasis prevalence, in primary school children on the shoreline of Lake Malawi, Mangochi District, Malawi

**DOI:** 10.1186/s40249-020-00736-w

**Published:** 2020-08-31

**Authors:** Sekeleghe A. Kayuni, Angus M. O’Ferrall, Hamish Baxter, Josie Hesketh, Bright Mainga, David Lally, Mohammad H. Al-Harbi, E. James LaCourse, Lazarus Juziwelo, Janelisa Musaya, Peter Makaula, J. Russell Stothard

**Affiliations:** 1 Department of Tropical Disease Biology, Liverpool School of Tropical Medicine, L3 5QA, Liverpool, UK; 2 Medi Clinic Limited, Medical Aid Society of Malawi (MASM), 22 Lower Sclatter Road, P.O. Box 1254, Blantyre, Malawi; 3 Laboratory Department, Mangochi District Hospital, P.O. Box 42, Mangochi, Malawi; 4 Malawi Liverpool Wellcome Trust Programme of Clinical Tropical Research, Queen Elizabeth Central Hospital, College of Medicine, P.O. Box 30096, Blantyre, Malawi; 5 Ministry of Health, Qassim, Kingdom of Saudi Arabia; 6 National Schistosomiasis and STH Control Programme, Ministry of Health, Lilongwe, Malawi; 7 Department of Basic Medical Sciences, College of Medicine, University of Malawi, Blantyre, Malawi; 8 Research for Health Environment and Development, P.O. Box 345, Mangochi, Malawi

**Keywords:** Emergence, *Schistosoma mansoni*, Urine CCA-dipstick, Faecal occult blood, Co-infection, Morbidity, COVID-19

## Abstract

**Background:**

Intestinal schistosomiasis was not considered endemic in Lake Malawi until November 2017 when populations of *Biomphalaria pfeifferi* were first reported; in May 2018, emergence of intestinal schistosomiasis was confirmed. This emergence was in spite of ongoing control of urogenital schistosomiasis by preventive chemotherapy. Our current study sought to ascertain whether intestinal schistosomiasis is transitioning from emergence to outbreak, to judge if stepped-up control interventions are needed.

**Methods:**

During late-May 2019, three cross-sectional surveys of primary school children for schistosomiasis were conducted using a combination of rapid diagnostic tests, parasitological examinations and applied morbidity-markers; 1) schistosomiasis dynamics were assessed at Samama (*n* = 80) and Mchoka (*n* = 80) schools, where *Schistosoma mansoni* was first reported, 2) occurrence of *S. mansoni* was investigated at two non-sampled schools, Mangochi Orphan Education and Training (MOET) (*n* = 60) and Koche (*n* = 60) schools, where *B. pfeifferi* was nearby, and 3) rapid mapping of schistosomiasis, and *B. pfeifferi*, conducted across a further 8 shoreline schools (*n* = 240). After data collection, univariate analyses and Chi-square testing were performed, followed by binary logistic regression using generalized linear models, to investigate epidemiological associations.

**Results:**

In total, 520 children from 12 lakeshore primary schools were examined, mean prevalence of *S. mansoni* by ‘positive’ urine circulating cathodic antigen (CCA)-dipsticks was 31.5% (95% confidence interval [*CI*]: 27.5–35.5). Upon comparisons of infection prevalence in May 2018, significant increases at Samama (relative risk [RR] = 1.7, 95% *CI*: 1.4–2.2) and Mchoka (RR = 2.7, 95% *CI*: 1.7–4.3) schools were observed. Intestinal schistosomiasis was confirmed at MOET (18.3%) and Koche (35.0%) schools, and in all rapid mapping schools, ranging from 10.0 to 56.7%. Several populations of *B. pfeifferi* were confirmed, with two new eastern shoreline locations noted. Mean prevalence of urogenital schistosomiasis was 24.0% (95% *CI*: 20.3–27.7).

**Conclusions:**

We notify that intestinal schistosomiasis, once considered non-endemic in Lake Malawi, is now transitioning from emergence to outbreak. Once control interventions can resume after coronavirus disease 2019 (COVID-19) suspensions, we recommend stepped-up preventive chemotherapy, with increased community-access to treatments, alongside renewed efforts in appropriate environmental control.

## Background

Lake Malawi is the world’s fourth largest freshwater lake, an important aquatic hotspot of global biodiversity but with urogenital schistosomiasis being endemic along many parts of its shoreline [1]. In Mangochi District, Malawi, the prevalence of *Schistosoma haematobium* infection in school children warrants preventive chemotherapy. This is achieved by annual mass drug administration (MDA) of praziquantel [2] as provided by the Malawi National Schistosomiasis and Soil-Transmitted Helminthiasis Control Programme (https://www.health.gov.mw/index.php/schistosomiasis-sth-control-programme). MDA is typically guided upon country-wide mapping information which is usually developed from inspection of five schools per district [3]. By contrast, intestinal schistosomiasis, caused by *Schistosoma mansoni*, is not considered endemic within the lake, as being congruent with the absence of *Biomphalaria pfeifferi*, an obligatory intermediate snail host and keystone snail species for parasite transmission [1, 4, 5].

This appraisal was revised in May 2018 as, since November 2017 *B. pfeifferi* has been repeatedly encountered in the lake, alongside emergence of intestinal schistosomiasis documented in three local primary schools [6]. Prevalence of infection by ‘trace/positive’ urine circulating cathodic antigen (CCA)-dipsticks was 34.3% (95% *CI*: 27.9–41.3), with ova-patent *S. mansoni* in stool noted at Samama and Mchoka schools [6]. Even with ongoing annual MDA for urogenital schistosomiasis control, the dynamics of intestinal schistosomiasis need further scrutiny here, for this disease could transition from emergence to outbreak.

Transitions from emergence to outbreak are often driven by expansions in the distributions of intermediate snail hosts which, like elsewhere in Africa, can instigate, for example, new transmission foci [7]. Even though an outbreak terminology is rather vaguely defined, common with the epidemiology of other water-borne diseases [8], it is more so for schistosomiasis as its transmission dynamics also involve unsafe water contact, with per-cutaneous (and oral) entry and infection routes. However, the use of outbreak vernacular can be appropriate, foremost, to spur commensurate public health actions, for example in stepped-up surveillance for the intermediate hosts or with intensified control interventions. This was evidenced in Senegal for intestinal schistosomiasis [9] and more recently in Corsica for urogenital schistosomiasis [10] which were each urged by the use of outbreak terminologies.

To seek an appropriate public health response here on the shoreline of Lake Malawi, our investigation had three linked objectives: 1) to resample Samama and Mchoka schools, ascertaining the dynamics of schistosomiasis infection and morbidity after annual MDA, 2) to confirm intestinal schistosomiasis, also noting faecal occult blood (FOB), at two previously non-sampled schools, Mangochi Orphan Education and Training (MOET) and Koche schools, where in 2018 *B. pfeifferi* was found nearby and 3) to conduct a wider rapid mapping survey for schistosomiasis at eight further schools (St Augustine II, Ndembo, Chikomwe, Chipeleka, Sungusya, St Martins, Makumba and Mtengeza) to judge if an outbreak of intestinal schistosomiasis was occurring.

## Methods

### Study design and sample size determination for each objective

A cross-sectional study design was used to achieve the three study objectives, see STROBE checklist within supplemental materials. Based on previous epidemiological information [6], a sample size calculation with single population proportion formula (http://www.raosoft.com/samplesize.html) showed that a total sample size of 520 was sufficient to estimate overall prevalence of intestinal and urogenital schistosomiasis with < ± 5% precision and 95% confidence.

**Fig. 1:**
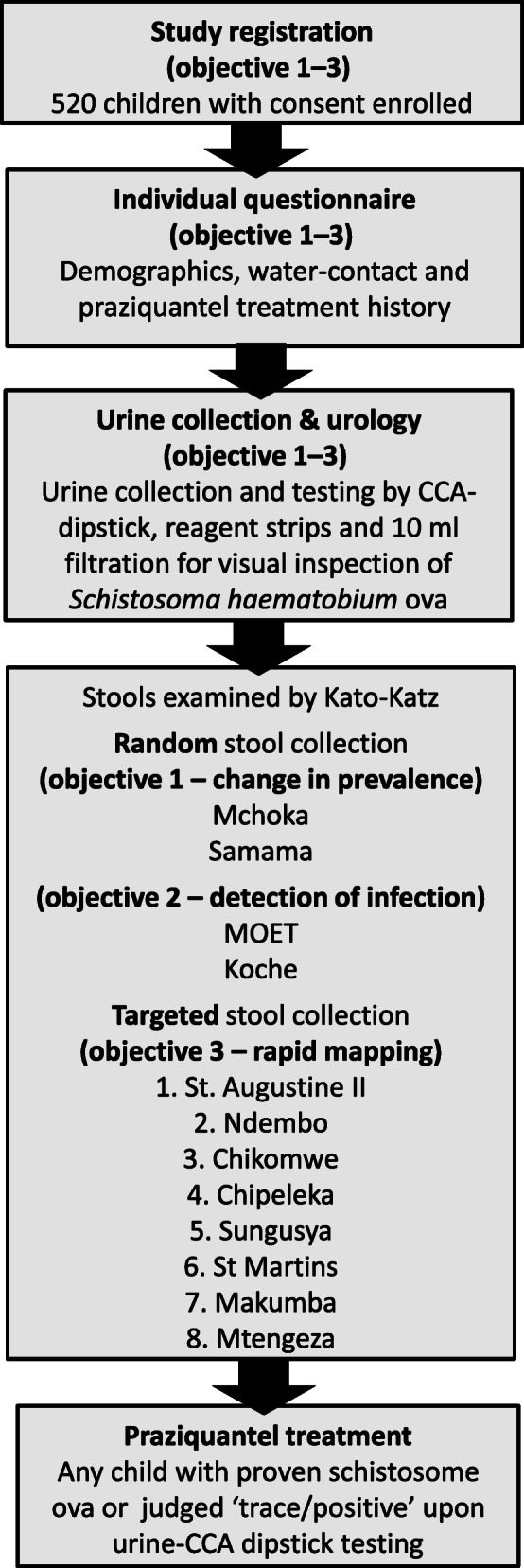
A study flow-chart of the objectives, sample size and methods used during this investigation

Based on prevalence data provided by the authors of the May 2018 study [6], a Fisher’s exact test was used to show that sampling of 80 children from each of Samama and Mchoka schools in June 2019 was sufficient to detect a 25-percentage-point rise in prevalence of each *Schistosoma* species at each school (α <  0.05, β <  0.20) (objective 1). To ensure detection of *S. mansoni* if present at MOET and Koche schools (objective 2), 60 children were sampled from each. For objective 3, according to World Health Organisation (WHO) recommendations for rapid mapping, 30 children per school were sampled per school [11]. Random sampling was used at each school following stratification by age and gender. A study flow diagram is included (see Fig. 1).

### Study area

**Fig. 2:**
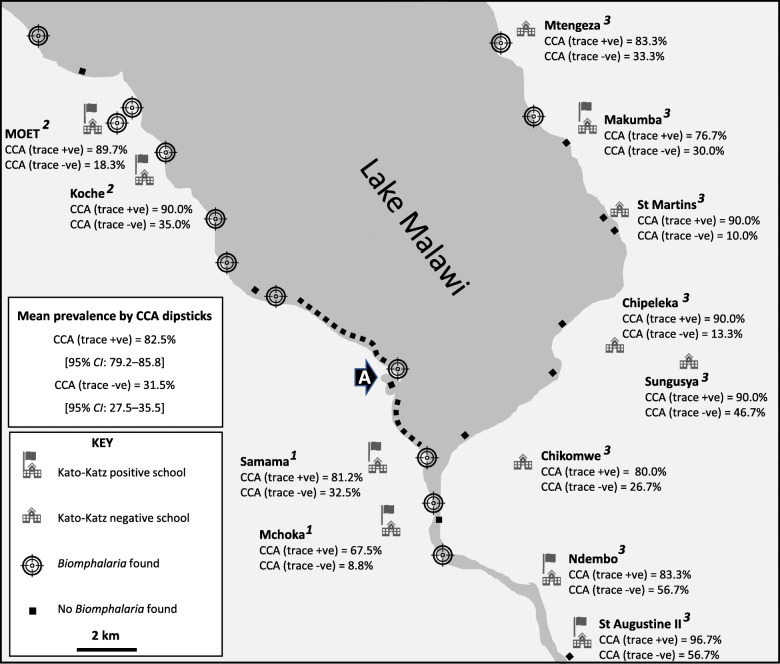
Schematic map showing the prevalence of intestinal schistosomiasis in June 2019, by sampled school, and by urine CCA-dipsticks Freshwater sites inspected for *Biomphalaria pfeifferi* over the November 2017–December 2019 period are also shown [**Note** that schools denoted with a flag represent locations where ova-patent *Schistosoma mansoni* infection was observed, and the schools associated with objectives 1–3. The black arrow labelled ‘A’ denotes the bay area as shown in Fig. 4 where the shoreline has changed during the 2005–2016 period, most likely due to lowering lake levels and local sedimentation, where numerous *B. pfeifferi* (*n* ≥ 10) have been consistently found]. +ve: positive; −ve: negative

At each school, global position system (GPS) coordinates were taken using an Oregon 650 receiver (Garmin, Olathe, Kansas, USA). The GPS locations for each school in decimal degrees are as follows: Samama (− 14.417465^o^, 35.217580^o^), Mchoka (− 14.439481^o^, 35.220644^o^), MOET (− 14.320776^o^, 35.131558 ^o^), Koche (− 14.330917^o^, 35.146186^o^), St Augustine II (− 14.473926^o^, 35.279613^o^), Ndembo (− 14.456385^o^, 35.273794^o^), Chikomwe (− 14.422136^o^, 35.265088^o^), Chipeleka (− 14.385387^o^, 35.292935^o^), Sungusya (− 14.386472^o^, 35.311398^o^), St Martins (− 14.351401^o^, 35.294435^o^), Makumba (− 14.319806^o^, 35.286104^o^) and Mtengeza (− 14.288932^o^, 35.264073^o^). A location map of the 12 schools is shown (see Fig. 2).

### Inclusion/exclusion criteria, diagnostics and praziquantel treatment of participants

The surveys took place during late May/June 2019; after obtaining written informed parental consent for each child, a total of 520 children, aged 6–15, of balanced gender, were enrolled. Children not attending school and acutely unwell children were excluded. Participants could withdraw consent at any point. On the appointed day of survey, each school child provided a mid-morning urine sample and when requested, a stool sample, alongside undertaking a brief interview by questionnaire documenting place of birth, recent travel, water-contact habits and praziquantel treatment history. If found infected, upon ova patent infection or ‘trace/positive’ urine CCA-dipstick test, each child was provided with praziquantel (IDA Foundation, Amsterdam, The Netherlands) at 40 mg/kg.

For detection of intestinal schistosomiasis, two drops of urine were applied to a CCA-dipstick (Rapid Medical Diagnostics, Pretoria, South Africa). Results were scored visually against a reference colour photograph as ‘negative’, ‘trace’ or ‘positive’ and cross-checked [12]. To augment urine CCA-dipsticks, on-site inspection of collected stool was performed with parasitological methods; at Mchoka, Samama, MOET and Koche schools, all children were asked to provide a stool sample with a total of 265 specimens obtained (see Table 1). Following our rapid mapping protocol at 8 remaining schools, stool was only requested from urine CCA-dipstick ‘positive’ children, obtaining 70 specimens (see Table 1).

**Table 1:** Occurrence of ova-patent *Schistosoma mansoni* in stool and prevalence and intensity of *S. haematobium* infections by school

School (sample size)	Stool: Kato-Katz (*S. mansoni*)					Urine: filtration (*S. haematobium*)		
	Number of stool samples collected	Prevalence (%) [95% *CI*]	Infection intensity^α^ (eggs per gram) [% of positives]			Prevalence (%) [95% *CI*]	Infection intensity^β^ (eggs per 10 ml) [% of positives]	
			Light	Medium	Heavy		Light	Heavy
TOTAL (*n* = 520)	335	*	20 [74.1]	4 [14.8]	3 [11.1]	24.0 [20.3–27.7]	80 [64.0]	45 [36.0]
All collected stools, irrespective of CCA status, were examined								
Mchoka (*n* = 80)	73	1.4 [0.0–4.1]	1 [100.0]	0 [0.0]	0 [0.0]	18.8 [10.2–27.4]	11 [73.3]	4 [26.7]
Samama (*n* = 80)	77	5.2 [0.2–10.2]	4 [100.0]	0 [0.0]	0 [0.0]	56.3 [45.4–67.2]	30 [66.7]	15 [33.3]
MOET (*n* = 60)	56	3.6 [0.0–8.5]	2 [100.0]	0 [0.0]	0 [0.0]	8.3 [1.3–15.3]	3 [60.0]	2 [40.0]
Koche (*n* = 60)	59	15.3 [6.1–24.5]	3 [33.3]	3 [33.3]	3 [33.3]	1.7 [0.0–5.0]	1 [100.0]	0 [0.0]
Only selective stools from urine-CCA ‘positive’ children were examined								
St Augustine II (*n* = 30)	15	*	3 [100.0]	0 [0.0]	0 [0.0]	43.3 [25.6–61.0]	8 [61.5]	5 [38.5]
Ndembo (*n* = 30)	15	*	6 [85.7]	1 [14.3]	0 [0.0]	60.0 [42.5–77.5]	7 [38.9]	11 [61.1]
Chikomwe (*n* = 30)	10	*	0 [−]	0 [−]	0 [−]	10.0 [0.0–20.7]	1 [33.3]	2 [66.7]
Chipeleka (*n* = 30)	3	*	0 [−]	0 [−]	0 [−]	26.7 [10.9–42.5]	4 [50.0]	4 [50.0]
Sungusya (*n* = 30)	7	*	0 [−]	0 [−]	0 [−]	16.7 [3.4–30.0]	4 [80.0]	1 [20.0]
St Martins (*n* = 30)	4	*	0 [−]	0 [−]	0 [−]	3.3 [0.0–9.7]	1 [100.0]	0 [0.0]
Makumba (*n* = 30)	6	*	1 [100.0]	0 [0.0]	0 [0.0]	6.7 [0.0–15.6]	2 [100.0]	0 [0.0]
Mtengeza (*n* = 30)	10	*	0 [−]	0 [−]	0 [−]	30.0 [13.6–46.4]	8 [88.9]	1 [11.1]

* unable to report prevalence due to selective stool sampling (8.1% of total stool collected was ova-patent; 15.7% of stool collected in selective sampling was ova-patent)

^α^ intensity by Kato-Katz: light: 1–99 epg; medium: 100–399 epg; heavy: ≥ 400 epg

^β^ intensity by urine filtration: light: < 50 ova per 10 ml; heavy: ≥ 50 ova per 10 ml

- calculation not applicable

To visualize helminth ova in stool, individual specimens were filtered across a 212 μm metal mesh then applied to produce duplicate thick (41.7 mg) Kato-Katz [11] smears as examined for lateral spine *S. mansoni* ova by microscopy (× 100). Intensity of *S. mansoni* infection as eggs per gram (epg) was classified as: light (1–99 epg), medium (100–399 epg) and heavy (≥ 400 epg) according to WHO guidelines [11]. To assess putative pathology associated with intestinal schistosomiasis [5], stools were screened for FOB using ALLTEST® cassettes (Access Diagnostic Tests UK Ltd., Aylsham, UK).

For detection of urogenital schistosomiasis, 10 ml of well-mixed urine was filtered by syringe across a circular nylon mesh of 1.5 cm diameter, with 20 μm pore size (Plastok® [Meshes and Filtration] Ltd., Birkenhead, UK). The mesh was stained with Lugol’s iodine, then inspected by microscopy (× 100) to count terminal spined *S. haematobium* ova. Infection intensity was classified as light (< 50 ova per 10 ml) or heavy (≥ 50 ova per 10 ml) according to WHO guidelines [11]. Putative pathology associated with urogenital schistosomiasis was assessed by Siemens Multistix® 10 SG reagent strips (Medisave UK Ltd., Weymouth, UK) for microhematuria [5].

### Malacological surveillance

During May/June 2019, all known locations where *B. pfeifferi* was found were re-surveyed, alongside several new locations as visited on the eastern shoreline of the lake, based upon convenience sampling from in-field observations of human water contact. At each site, two collectors searched, for 20 min, for *B. pfeifferi* by hand and with metal sieves. GPS coordinates, altitude and location photographs were taken with an Oregon 650 receiver (Garmin, Olathe, Kansas, USA). Water temperature (°C), pH and conductivity (μS) were recorded with a HI-98129 Pocket EC/TDS and pH Tester (Hanna Instruments Ltd., Leighton Buzzard, Bedfordshire, UK)*.* Collected snails were kept for a week and screened daily for shedding *S. mansoni* cercariae by exposure to sunlight under a dissecting microscope (× 20).

### Data analyses

Demographic, questionnaire and diagnostic data were tabulated with statistical analysis carried out using IBM SPSS® Version 24 (IBM, Portsmouth, UK). Univariate analyses and Chi-square testing were first performed, then binary logistic regression undertaken, calculating adjusted odds ratios with generalised linear models, with stepwise subtraction of variables, to investigate (un)adjusted epidemiological associations.

## Results

### Prevalence and distribution of intestinal and urogenital schistosomiasis

The outline map, Fig. 2, is a summary of all information obtained from urine CCA-dipsticks with the distribution of intestinal schistosomiasis displayed. When ‘trace’ was considered infected, mean prevalence was 82.5%. When ‘trace’ was considered not infected, this declined to 31.5%. Common across all school children were very high levels of reported weekly water contact (> 75%), inclusive of bathing, swimming and drinking. The known distribution of *B. pfeifferi* along the western shoreline, alongside new reports on the eastern shoreline in December 2018 and May/June 2019, is shown. In locations where *B. pfeifferi* was found, water parameters ranged: pH 7.5–8.5, temperature 21.5–26.2 °C, conductivity 312–458 μS and total dissolved salts 155–244 ppm; no collected snail (*n* = 52) was observed to shed *S. mansoni* cercariae.

Ova-patent *S. mansoni* infections, including both medium and heavy intensity infections, were observed (see Table 1). Ova patent urogenital schistosomiasis was detected in all schools, ranging from 1.7 to 60.0%, inclusive of heavy intensity infections, except at Koche, St Martins and Makumba schools. Across our sample, 75 (14.4%) children were considered ‘free’ from schistosomiasis; if urine CCA-dipstick ‘trace’ was considered infected or ‘trace’ was considered not infected, then 109 (36.5%) or 56 (10.7%) children were judged co-infected with intestinal and urogenital schistosomiasis, respectively.

### Risk factors associated with schistosomiasis-associated morbidity

**Fig. 3:**
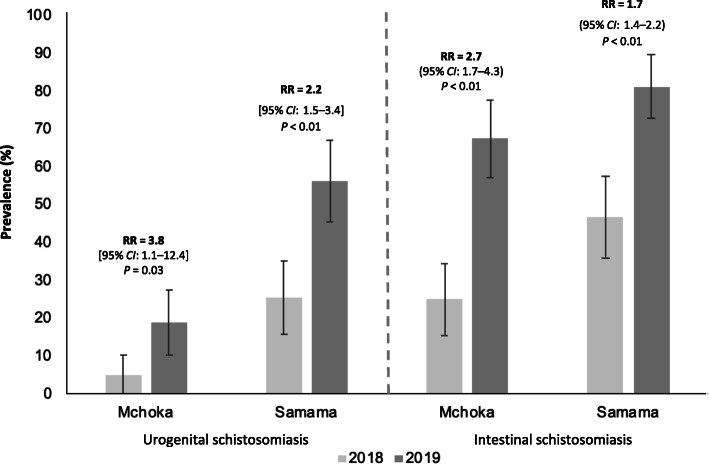
The year-on-year increase of prevalence of urogenital (by urine filtration) and intestinal (by ‘trace/positive’ urine CCA-dipsticks) schistosomiasis despite annual MDA across the two schools Mchoka and Samama as sampled in 2018 and 2019. Error bars indicate 95% confidence intervals (*CI*). RR: relative risk

Significant increases of schistosomiasis at Mchoka and Samama were observed (see Fig. 3) even though MDA treatment coverage (81.9%), as reported by interview, was good. Relative risk of infection prevalence of *S. mansoni* significantly increased at Samama (RR = 1.7, 95% *CI*: 1.4–2.2) and Mchoka (RR = 2.7, 95% *CI*: 1.7–4.3) schools, indicative of substantive re-infection concurrent with increasing environmental transmission for both types of schistosomiasis.

Analysis of risk factors associated with schistosomiasis-associated morbidity (see Table 2) showed that ‘positive’ urine CCA-dipstick results and ova-patent *S. mansoni* were significantly associated with FOB, alongside ova-patent *S. haematobium* with microhaematuria. Neither age nor gender were associated with these morbidity indicators although a marginal protective effect of MDA, on both FOB and microhaematuria, was observed.

**Table 2:** Risk factors analyses for morbidity associated with urogenital and intestinal schistosomiasis upon detection of microhematuria and faecal occult blood, respectively

Prevalence (%) [95% *CI*]		Microhematuria		Faecal occult blood (FOB)	
		31.5 [27.5–35.5]		16.2 [11.0–21.4]	
Sample size		*n* = 520		*n* = 191^α^	
		Unadjusted odds ratio (95% ***CI***) [***P***-value]	Adjusted odds ratio (95% ***CI***) [***P***-value]	Unadjusted odds ratio (95% ***CI***) [***P***-value]	Adjusted odds ratio (95% ***CI***) [***P***-value]
Urine-CCA test^β^	Negative	1	1	1	1
	Positive	2.0 (1.4–3.0) [< 0.01]	1.2 (0.6–2.6) [0.61]	12.9 (4.3–38.7) [< 0.01]	9.2 (3.0–28.6) [< 0.01]
Ova-patent intestinal schistosomiasis (Kato-Katz)	Negative	1	1	1	1
	Positive	2.2 (1.0–4.7) [0.06]	3.0 (1.0–8.6) [0.04]	11.4 (3.9–33.3) [< 0.01]	6.7 (2.0–22.6) [< 0.01]
Ova-patent urogenital schistosomiasis (urine filtration)	Negative	1	1	1	1
	Positive	42.1 (23.2–76.5) [< 0.01]	47.9 (22.6–101.5) [< 0.01]	1.6 (0.7–3.8) [0.25]	1.5 (0.5–4.9) [0.49]
Praziquantel treatment in last 12 months	No	1	1	1	1
	Yes	0.7 (0.5–1.1) [0.16]	0.7 (0.3–1.8) [0.45]	0.5 (0.2–1.3) [0.16]	0.8 (0.3–2.3) [0.65]
Gender	Male	1	1	1	1
	Female	1.0 (0.7–1.4) [0.85]	0.9 (0.5–1.8) [0.82]	1.1 (0.5–2.3) [1.00]	1.0 (0.4–2.4) [0.97]
Age (years)	6–10	1	1	1	1
	11–15	0.9 (0.6–1.4) [0.71]	1.2 (0.6–2.3) [0.63]	1.1 (0.507–2.4) [0.81]	0.9 (0.3–2.3) [0.78]

^α^ all total of 200 FOB tests were available being used at Samama, Mchoka and MOET schools;

^β^ a trace result was considered here as not infected, only + ve urine CCA-dipstick scorings were considered infected; our conservative approach was based upon correlates of urine CCA-dipsticks and duplicate Kato-Katz comparisons, with ova-patent prevalence of *S. mansoni* being ≥ 20%, see Bärenbold et al. [12]

## Discussion

Our integrated surveillance approach was unified by three linked cross-sectional surveys, see Fig. 1, and a conjoined malacological inspection. Collectively this builds a more thorough assessment of the changing epidemiology of intestinal and urogenital schistosomiasis on the Lake Malawi shoreline (see Fig. 2 and Table 1). Of note, is that the prevalence of both forms of schistosomiasis is increasing (see Fig. 3), indicative perhaps that the force of infection [13] for each parasite is rising, with intestinal schistosomiasis being of newest public health concern here.

Our study detected a mean prevalence of intestinal schistosomiasis by ‘positive’ urine CCA-dipstick results of 31.5% (95% *CI*: 27.5–35.5). Notably, significant increases in infection prevalence since May 2018 were observed at Samama (RR = 1.7, 95% *CI*: 1.4–2.2) and Mchoka (RR = 2.7, 95% *CI*: 1.7–4.3) schools. The disease was also confirmed at MOET (18.3%) and Koche (35.0%) schools with a broader geographical footprint apparent across the 8 rapid mapping schools, with prevalence ranging from 10.0 to 56.7%, and several extant populations of *B. pfeifferi* were confirmed on the eastern and western lake shoreline. Concurrently mean prevalence of urogenital schistosomiasis was 24.0% (95% *CI*: 20.3–27.7) with 109 (36.5%) or 56 (10.7%) children co-infected with intestinal schistosomiasis, as contingent upon interpretation of urine-CCA dipstick ‘trace’ as infection-positive or negative, respectively.

The unexpected occurrence of intestinal schistosomiasis elsewhere in Malawi, alongside the more well-known urogenital schistosomiasis, has been encountered before; the surveys conducted by Poole et al. in Chikhwawa during June 2012 noted that 24.9 and 9.1% of mothers and their pre-school-aged children were positive by urine CCA-dipsticks with ova-patent *S. mansoni* infections confirmed [14]. While *Biomphalaria* was not detected in their search for local snails [14], the occurrence of *B. pfeifferi*, as shown here in Fig. 2, adds weight to their postulate of intermittent transmission of *S. mansoni* in Chikhwawa. They suggested that the occasional influx of upstream populations of *B. pfeifferi* in the Shire River, as being swept downstream during seasonal flooding, might then colonize temporary pools in the Lower Shire River flood plain, to spark sporadic transmission in Chikhwawa [14]. By contrast, an enduring presence of *B. pfeifferi* along Lake Malawi and Upper Shire River, gives rise to more sustained opportunities in local transmission of *S. mansoni* in Mangochi District.

In regard of this lake shoreline setting, we have shown 1) increases in the prevalence of intestinal schistosomiasis at Mchoka and Samama schools, 2) occurrence of intestinal schistosomiasis at MOET and Koche schools and 3) endemic intestinal schistosomiasis occurring along a 80 km section of Lake Malawi and Shire River shoreline, noting additional populations of *B. pfeifferi* on the lake’s eastern shoreline (see Fig. 2). Of particular note is the strong association of *S. mansoni* infection, as detected by urine CCA-dipsticks, with FOB in 16.2% of examined children, see Table 2, indicative of overt intestinal pathology [15]. Combined with the observations of ova-patent infections of moderate- and heavy-intensities at Koche and Ndembo, as well as, ova-patent infections at a further five schools, this is pervasive evidence of more sustained local transmission of intestinal schistosomiasis.

Whilst the debate on how to interpret ‘trace’ reactions of urine-CCA dipsticks continues, a ‘positive’ reaction is considered solid evidence of active intestinal schistosomiasis [12]. With no association detected between urogenital schistosomiasis and urine-CCA in our study, we conclude that urine-CCA tests are highly specific for *S. mansoni* detection, with ‘trace’ results indicating light sub-clinical infections, with sub-patent egg outputs. Therefore, 31.5% (95% *CI*: 27.5–35.5) of our sampled children were suffering from intestinal schistosomiasis but if a ‘trace’ reaction was considered diseased then a total of 82.5% (95% *CI*: 79.2–85.8) were infected or, at the very least, at-risk. Of particular note in this light is intestinal schistosomiasis at Ndembo and St Augustine II schools, see Fig. 2, where the prevalence of ‘positive’ urine-CCA dipsticks was > 50% and ova-patent *S. mansoni* infections were encountered, being of light and moderate infection intensities, Table 1; moreover, moderate and heavy ova-patent *S. mansoni* infections were detected at Koche school where the prevalence of ‘positive’ urine-CCA dipsticks was 35.0%, with *B. pfeifferi* found nearby.

Our rapid disease mapping surveillance across eight schools, currently augments district-level information of the national control programme, critically revising scientific appraisals concerning the previous absence of intestinal schistosomiasis [1], and better demonstrates the newly defined endemicity of intestinal schistosomiasis along the Mangochi District shoreline. When taken as a whole, we judge that there is now sufficient evidence to notify that an outbreak of intestinal schistosomiasis is occurring. This has immediate bearing on the health of the local populace and tourists who may visit here, as well as, in health advice or diagnostic testing undertaken in local or international medical clinics presently unaware of this new risk of intestinal schistosomiasis.

**Fig. 4:**
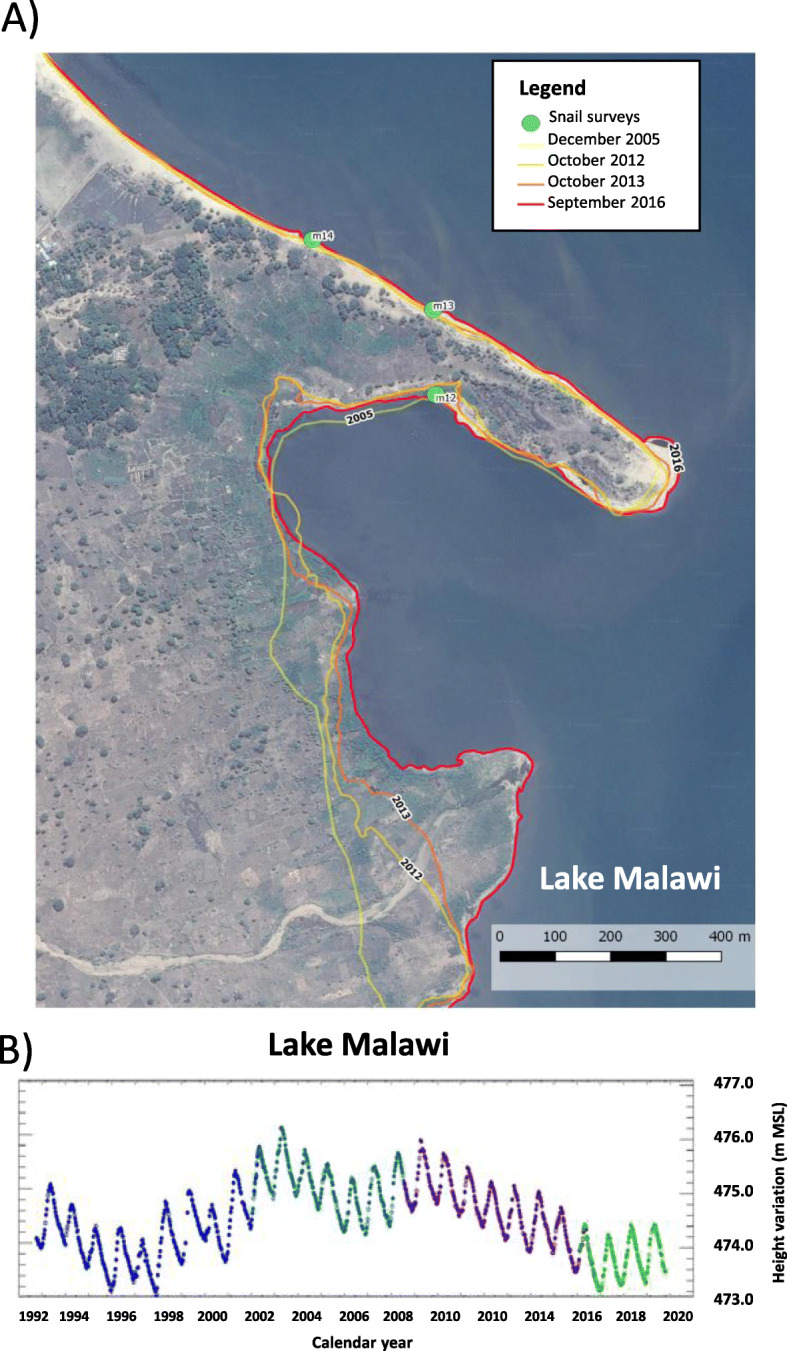
**a** Composite satellite map, modified from GoogleEarth imagery, that illustrates the changing shoreline of the lake in 2005, 2012, 2013 and 2016. The featured area is the bay indicated by the black arrow labelled ‘A’ in Fig. 1. The green circle ‘M12’ denotes the sampling location where numerus *Biomphalaria* have been found during all malacological inspections from November 2017 to December 2019. The changing shoreline is most likely resultant from lowering lake levels, see **b**, as well as, upon influx of sediments from the seasonal river in the bottom part of this image. **b**. Annual changes in the lake surface levels during 1992–2019 period (see https://ipad.fas.usda.gov/cropexplorer/global_reservoir/gr_regional_chart.aspx?regionid = eafrica&reservoir_name = Malawi), as detected by remote altimetry, denoting two particularly low-level periods, in 1996–1998 and 2017–2019, which may help explain the changing shoreline shown in **a** as the lake recedes in depth. m MSL: meters above Mean Sea Level

In terms of environmental surveillance, it is worthy to note that the lake is undergoing ecological change, most easily seen with lake level changes through time, see Fig. 4. Its dynamic shoreline and lake level are manifest, perhaps creating new habitats for *B. pfeifferi* to colonize and or were facilitating collection of this snail in locations previously too deep to be retrieved by hand. The dispersion of this snail, a keystone species for *S. mansoni*, like in Senegal [9] or in Ethiopia [7], is a critical epidemiological driver of intestinal schistosomiasis transmission.

Control of schistosomiasis needs a multisectoral approach and it is often debated how control tactics should be changed [16] or better tailored to aquatic environments [17]. To respond to this outbreak of intestinal schistosomiasis, we propose that current MDA efforts should be intensified, adopting biannual treatment cycles in schools, which has been successfully implemented elsewhere [18], alongside expanded access to praziquantel for all community members with intestinal schistosomiasis, in need of regular treatment throughout the year [19]. From recent surveys of adult fishermen who have urogenital schistosomiasis, making specific reference to male genital schistosomiasis, co-infection with *S. mansoni* has been noted alongside re-infections within a calendar year [20, 21]. To augment MDA and community-access to praziquantel, it is important to strengthen health education and outreach with suitable water, sanitation and hygiene (WASH) interventions [20, 22], better appropriate to this lakeshore setting, noting that even focal application of molluscicides is inappropriate [17], given this lake’s global importance in biodiversity.

A significant limitation of our study was the exclusion of certain demographic groups in our survey. This included pre-school-aged and out-of-school children, as well as, more vulnerable adults [23]. However, with increased future resourcing inspection of these groups is important to better assess how they are each afflicted by this outbreak. To do so, we recommend a combination of both rapid urine and faecal sampling methods with inspection of a more extensive range of point-of-contact morbidity markers to provide a better insight into individual disease progression(s) [24]. Future use of 20 m shuttle-run tests to assess children’s aerobic capacity in relation to *S. mansoni* infection could be insightful, as recently shown elsewhere [25]. However, with coronavirus disease 2019 (COVID-19) suspending annual MDA, we should expect and better prepare for increasing severity of intestinal schistosomiasis in following years.

## Conclusions

Our three main study objectives were achieved: demonstration of increasing prevalence of intestinal and urogenital schistosomiasis at Samama and Mchoka schools, newly confirmed intestinal schistosomiasis at previously non-sampled schools near reported *B. pfeifferi* sites (MOET and Koche schools), and detection of intestinal schistosomiasis at a further eight sampled schools along the shoreline. Despite ongoing annual MDA of praziquantel for urogenital schistosomiasis, we conclude that an outbreak of intestinal schistosomiasis is occurring in Mangochi District, Malawi. Increased vigilance for *B. pfeifferi*, especially along the lake’s eastern shores and in downstream locations on the Shire River, is needed with additional epidemiological inspections of adjacent schools and communities to better gauge the full footprint of intestinal schistosomiasis. Due to the COVID-19 pandemic, this outbreak will continue to expand unchecked, but once control activities can resume, we strongly recommend stepping-up MDA treatment cycles, i.e. from annual to biannual, increasing community access to praziquantel treatment throughout the year, with renewed efforts to mitigate environmental transmission with health education and appropriate WASH interventions.

## Data Availability

Data used for the analysis are available from the corresponding author upon reasonable request.

## Abbreviations

CCA: Circulating cathodic antigen
*CI*: Confidence interval
COVID-19: Coronavirus disease 2019
EPG: Eggs per gram
FOB: Faecal occult blood
GPS: Global positioning system
MDA: Mass drug administration
MOET: Mangochi Orphanage Education and Training
m MSL: meters above mean sea level
RR: Relative risk
WASH: Water, sanitation and hygiene
WHO: World Health Organisation
